# Effect of phytoplankton morphology on the measurement of biovolume

**DOI:** 10.1093/plankt/fbag011

**Published:** 2026-03-05

**Authors:** Christopher R Farrow, Josef D Ackerman

**Affiliations:** Physical Ecology Laboratory, Department of Integrative Biology, University of Guelph, 50 Stone Rd E, Guelph, ON N1G 2W1, Canada; Physical Ecology Laboratory, Department of Integrative Biology, University of Guelph, 50 Stone Rd E, Guelph, ON N1G 2W1, Canada

**Keywords:** imaging flow cytometry, image-based biovolume estimates, phytoplankton morphology, automatic sampling devices

## Abstract

Algal biovolume is a standard measurement for limnological and oceanographic studies, which is used to proxy biomass and productivity. Manual and automatic methods can be applied to estimate biovolume from phytoplankton images. We examined the effects of algal shape and taxon (largely genus) on the accuracy of automatic image-based biovolume estimates for samples from a large oligotrophic embayment (Georgian Bay, Lake Huron). Specifically, we compared biovolume estimates based on equivalent spherical diameter, Feret-based diameter and two shape-based estimates (cylinder and prolate spheroid) against the manual biovolume measurements using correlation, linear regression and analysis of variance. The automatic biovolume estimates were all moderately to strongly correlated with the manual measurements, but the automatic cylinder method was the most accurate, overall. The automatic cylinder method overpredicted the manual total biovolume measurement of the community by 59%, but it had the strongest linear relationship with manual total biovolume, and the strongest correlation based on the biovolumes of individual genera. When algal taxa were assigned to 11 general shape categories, we observed significant differences in the accuracy with which they were measured. Results suggest that the cylinder biovolume method provides a reasonable estimate of total biovolume in algal communities that have a diversity of elongated shapes.

## INTRODUCTION

The estimation of phytoplankton biovolume is a routine aspect of microscopic analyses of water samples for limnological (Canfield Jr. *et al.,* 2019) and oceanographic studies (Harrison *et al.,* 2015). Algal biovolume can be used to estimate biomass and productivity (LaBaugh, 1995). The standard approach for algal biovolume estimates involves the use of simple geometric shape formulae, often using a single shape for organisms with simple morphologies (e.g. bacterioplankton) or multiple combinations of shapes for organisms with more complex morphologies (e.g. eukaryotic phytoplankton) (Hillebrand *et al.,* 1999; Saccà, 2016, 2017). Although it is possible to obtain measurements of specific dimensions under a microscope, this is laborious work, which is outpaced by automated methods (Álvarez *et al.,* 2012, 2014). High volumes of image data and associated biovolume measurements can be obtained rapidly with automated sample processing methods such as imaging flow cytometry (Dashkova *et al.,* 2017).

Whether manual microscopy or an automated sample processing method is used, a collection of phytoplankton images is often obtained for later analysis. Morphometric data are extracted from the pixels in these images, which can be analyzed manually (e.g. Hillebrand *et al.,* 1999; Sun and Liu, 2003) or automatically (Sieracki *et al.,* 1989; Moberg and Sosik, 2012; Saccà, 2017). Biovolumes estimated from images obtained from specific taxa correlate with measurements taken directly under a microscope (Sieracki *et al.,* 1989; Moberg and Sosik, 2012; Saccà, 2017) but are subject to sources of error pertaining to the measurement of individual cells or other natural units (e.g. colonies or filaments). These sources of error include (i) poor image focus, (ii) the application of volume formulae that are not specific to a given taxonomic group or cells and (iii) image thresholding issues that affect the detection of cell boundaries. Despite these issues, imaging flow cytometers (e.g. FlowCam) can measure the size of individual diatom cells accurately (Spaulding *et al.,* 2012) and provide biovolume estimates that compare well with traditional microscopy for total biovolume (Álvarez *et al.,* 2014), as well as for specific taxonomic groups, in some cases (Hrycik *et al.,* 2019). In other cases, the biovolumes of certain taxa (e.g. long centric diatom chains) are measured inaccurately because of their distinct elongated shape (Álvarez *et al.,* 2012). High abundances of taxa with complex morphologies can reduce the accuracy of total (community) biovolume estimates (Álvarez *et al.,* 2012). Fortunately, research has examined the best method to estimate biovolume from a particular genus when the shape is known (Hrycik *et al.,* 2019) or has obtained information about the shape in order to apply the best method (Álvarez *et al.,* 2012, 2014). The most accurate method for biovolume estimation without knowledge of which shapes are present in the sample, however, remains unknown. This applies to biovolume estimates for select species or for broad taxonomic groups within a community, as well as uncategorized data collected at higher taxonomic resolutions (e.g. genus or species) for whole algal communities. Within broad taxonomic groups, it is possible that individual species or genera may be measured with different accuracies and that some automatic biovolume estimates may outperform others. To the best of our knowledge, this possibility has not been examined, but this information would be useful for algal community ecology as well as studies that focus on indicator taxa including Harmful Algal Blooms.

Given that cell or colony morphologies can vary within and among broad taxonomic groups (e.g. diatoms), the assignment of generalized shape designations (e.g. ellipsoid, sphere, cylinder; Menden-Deuer and Lessard, 2000) and the extrapolation of 2D image data to cell volumes (Owen *et al.,* 2022) are likely two of the most significant sources of error in image-based biovolume estimates. Regardless, two of the most widely used automatic methods involve the measurement of: (i) the area of a particle in an image to estimate diameter (equivalent spherical diameter [ESD]); or (ii) several Feret measurements to estimate diameter (Feret-based diameter [FBD]). Based on the underlying diameter estimate, both methods assume the volume of the particle is equal to that of a sphere of equivalent size. Both methods are sensitive to the shape of the particle and the presence of various transparent structures (Owen *et al.,* 2022), but the area-based method is understood to be the more accurate and preferred method of the two (Jakobsen and Carstensen, 2011). The specific circumstances in which to use one method over the other, or to use basic shape formulae instead, are unclear because assessments at levels of taxonomic resolution finer than class (e.g. diatoms) are limited. Moberg and Sosik (2012) identified certain genera (e.g. *Thalassiosira*) that cannot be measured accurately with computer algorithms, but it is unknown how measurement accuracies differ among genera within an algal community. Thus, it is important to identify the accuracy of automatic biovolume measurement methods in relation to specific phytoplankton morphologies, which vary substantially among and even within taxonomic categories (Ryabov *et al.,* 2021).

The purpose of this study is, therefore, to determine the effect of phytoplankton morphology on the measurement of total biovolume, as well as algal taxon- and shape-specific biovolumes derived from an automatic image-based sampling method. To accomplish this objective, we compared manual shape-based biovolume measurements with ESD biovolume, FBD biovolume and automatically calculated shape-based biovolumes (prolate spheroid and cylinder) taken by an imaging flow cytometer (FlowCam) for a diverse algal community characterized by 11 shapes (see Table I). We hypothesize that the accuracy of image-based biovolume measurements depends on algal shape because some cells are roughly equivalent in size in all three dimensions, whereas others are not. We, therefore, examine whether automatic measurements designed for specific algal shapes (e.g. cylinder method, prolate spheroid method) are more accurate than generalized volume estimates (i.e. equivalent spherical diameter- and Feret-based biovolume) when applied to cells of those respective shapes. Indeed, others have compared the accuracy of imaging flow cytometer-based biovolume measurements among broad taxonomic categories (Álvarez *et al.,* 2012, 2014), as well as among general algal shape categories (Hrycik *et al.,* 2019). The present study advances previous research by identifying whether variation among individual genera, which is not encompassed by broad taxonomic or shape categories, could affect the accuracies of total biovolume estimates. Moreover, it should provide information on the most accurate method for biovolume estimation when the knowledge of which algal shapes are present in the sample is unknown.

**Table I:** Eleven basic algal shape categories used to assign broad taxonomic categories. The asterisks indicate taxa that require at least two geometric shapes for manual biovolume analyses

Representative morphology and silhouette	Algal taxa	Broad taxonomic categories
(A) Irregular cluster 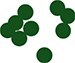	*Chroococcus*	Cyanobacteria
(B) Irregular dense 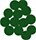	*Aphanocapsa, Aphanothece, Woronichinia* sp1, *Woronichinia* sp2	Cyanobacteria
(C) Rectangular colony with ellipsoidal cells 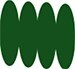	*Merismopedia, Desmodesmus, Scenedesmus*	Cyanobacteria, Chlorophyte
(D) Cylindrical 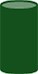	*Oscillatoria, Spirulina, Asterionella, Aulacoseira, Cyclotella, Melosira, Navicula, Nitzschia, Tabellaria,* unknown pennate diatoms, *Pediastrum*	Cyanobacteria, Diatoms, Chlorophyte
(E) Half paralleliped 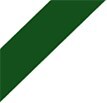	*Cymbella*	Diatom
(F) Rectangular colony with cylindrical cells 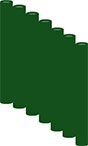	*Fragilaria capucina*, *Fragilaria crotonensis*	Diatom
(G) Sigmoidal 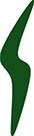	*Gyrosigma*	Diatom
(H) Conical 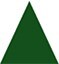	*Actinastrum, Ankistrodesmus, Closterium, Kirchneriella, Staurastrum, Tetradesmus*	Chlorophyte
(I) Ellipsoidal 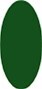	*Carteria*, Cosmarium^*^, Crucigenia, Oocystis, Chroomonas, Cryptomonas, unknown cryptophytes, *Dinobryon, Uroglena, Mallomonas, Synura, Euglena*	Chlorophyte, Cryptophyte, Chrysophyte, Synurophyte, Euglenophyte
(J) Spherical 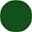	*Chlamydomonas*, *Coelastrum*, *Chlorella*, *Dictyospherium, Golenkinia, Micractinium, Sphaerocystis*	Chlorophyte
(K) Spheroidal/Conical 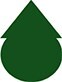	Peridinaceae^*^	Dinoflagellate

## METHODS

We used an existing analysis of 72 phytoplankton samples collected from a nearshore transect in Nottawasaga Bay, a large and open oligotrophic embayment of Georgian Bay, Lake Huron in 2016 (Farrow *et al.,* 2020). On average 1946 ± 202 cells were identified per sample from images obtained under 10 × magnification using a FlowCam VS-4 (Yokagawa Fluid Imaging Technologies, Inc.) operated in fluorescence trigger mode. This value corresponded to 19% of the imaged particles because cells less than 5 microns in size were not identified, as they occupied too few pixels under 10× to resolve differences in morphology. Nonetheless, they represented a significant portion of the fluorescent particles (Farrow *et al.,* 2020). A total of 50 unique phytoplankton taxa (mostly at the level of genus) from eight broad taxonomic categories (Table I) and with a diverse range of characteristic morphologies were identified. The biovolume of cells imaged by FlowCam were measured manually using Image J (Schneider *et al.,* 2012) because the built-in Visual Spreadsheet software (version 3.4.5) lacked the capability to measure particles within “image libraries” (i.e. a collection of images of a single algal taxon) at the time of the study. Hidden dimensions were estimated from published data (Olenina *et al.,* 2006; Napiorkówska-Krzebietke and Kobos, 2016). Simple geometric shape formulae (Olenina *et al.,* 2006; Brierley *et al.,* 2007) were applied to the average dimensions of 30 cells of each algal taxon, which were compiled from representative images across the 72 samples. This approach was used because it was not always possible to obtain 30 cells within a given sample. Thus, the average volume per cell, which was based on the results from 30 images, was multiplied by the number of cells in each sample to estimate the total biovolume for each taxon.

Visual Spreadsheet (version 5.9.0.74) extracts morphometric feature data from the collected images and calculates biovolume automatically using four methods, two using equivalent diameters (ESD and FBD) for spheres and two using shape-based (prolate spheroid, and cylinder) methods. The first method represents the particle as a sphere whose diameter is equal to the area-based diameter (ABD; referred to as “sphere” in Visual Spreadsheet). Before the particle can be represented in three dimensions, the number of dark pixels in the binary image is used to approximate the area of the particle, which uses a calibration factor specific to each instrument. The diameter of a circle with area equal to the ABD area is the “ESD” (referred to as “ABD” in Visual Spreadsheet), which is used in the final sphere volume calculation,


(1) \begin{equation*} volume\ (ESD)=\frac{4}{3}\pi{\left(\frac{ESD}{2}\right)}^3. \end{equation*}

The second volume estimate also models the particle as an equivalent sphere, but it uses length measurements. In this case, the diameter of the sphere is the Feret-based diameter (FBD), which is based on the average of 36 Feret diameter measurements taken at 5-degree intervals from −90° to 90° (referred to as “ESD” in Visual Spreadsheet). In this case, the volume is represented by


(2) \begin{equation*} volume\ (FBD)=\frac{4}{3}\pi{\left(\frac{FBD}{2}\right)}^3. \end{equation*}

The prolate spheroid volume is based on the equation,


(3) \begin{equation*} volume\ \left( prolate\ spheroid\right)=\frac{4}{3}\pi{w}^2l \end{equation*}

where $w$ is width and $l$ is length of the Legendre ellipse. The cylinder volume formula is based on the equation,


(4) \begin{equation*} volume\ (cylinder)={\pi r}^2h \end{equation*}

where $r$ is the radius and $h$ is the height. The radius is estimated as half the geodesic thickness, and the height as the geodesic length.

### Statistical analyses

We compared the accuracy of the automatic total biovolume measurements against the manual measurements using linear regression. Pearson correlation matrices were used to assess the correlations among biovolume measurements across all plankton samples and algal taxa. Linear regressions were used to determine whether shape specific biovolume estimates of certain algal taxa were measured more accurately than others. Specifically, we compared the coefficients of determination from these regressions among the 11 algal shape categories, which were comprised of various taxonomic groups (Table I). We used a two way ANOVA to determine which methods was/were more accurate based on their mean differences from the manual method for each of the 11 shape categories. Finally, we compared total biovolume of the algal community calculated by each method versus the manual value using ANOVA and a Tukey test for pairwise differences. This was undertaken to determine which automated biovolume method (ESD, FBD, cylinder method, prolate spheroid method) is most accurate. Assumptions of homogeneity of variance and normality of residuals were examined with Levene’s tests and Shapiro–Wilk tests, respectively.

An underlying assumption of the analyses described above is that the manual biovolume measurements were measured without error, given that cell measurements agree with conventional microscopy-based measurements (Spaulding *et al.,* 2012). Thus, we assumed that variation in regression coefficients is attributable predominantly to the automatic biovolume methods and the associated automatic extraction of morphometric data, rather than the FlowCam’s ability to measure cells.

## RESULTS

### Total biovolume

**Fig. 1:**
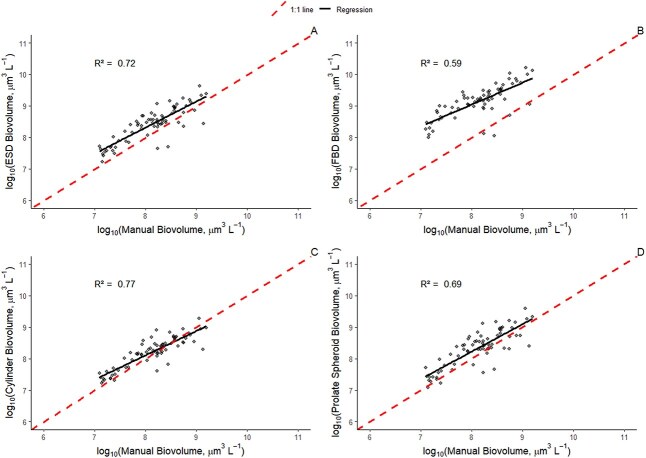
Linear regressions between (a) ESD biovolume, (b) FBD biovolume, (c) cylinder biovolume method and (d) prolate spheroid biovolume method and the manual biovolume measurements (n = 72 samples; 1946 ± 202 cells per sample; see Farrow *et al.,* 2020).

Manual biovolume measurements for cells or filaments (i.e. natural units) are presented in the Appendix (Table S1). All automatic methods were linearly related to the manual total biovolume measurements on a log–log scale (Fig. 1). The cylinder biovolume method (Fig. 1c) had the strongest association with manual total biovolume (F_1,70_ = 230, *R^2^* = 0.77, *P* < 0.001). ESD (F_1,70_ = 182, *R^2^* = 0.72, *P* < 0.001) and the prolate spheroid method (F_1,69_ = 151, *R^2^* = 0.69, *P* < 0.001) both explained a moderate amount of variation in manual total biovolume measurements. In contrast, FBD biovolume had the weakest association with the manual total biovolume (F_1,70_ = 100, *R^2^* = 0.59, *P* < 0.001) and overpredicted most of the manual measurements by approximately an order of magnitude.

### Taxon-specific biovolume correlations

**Fig. 2:**
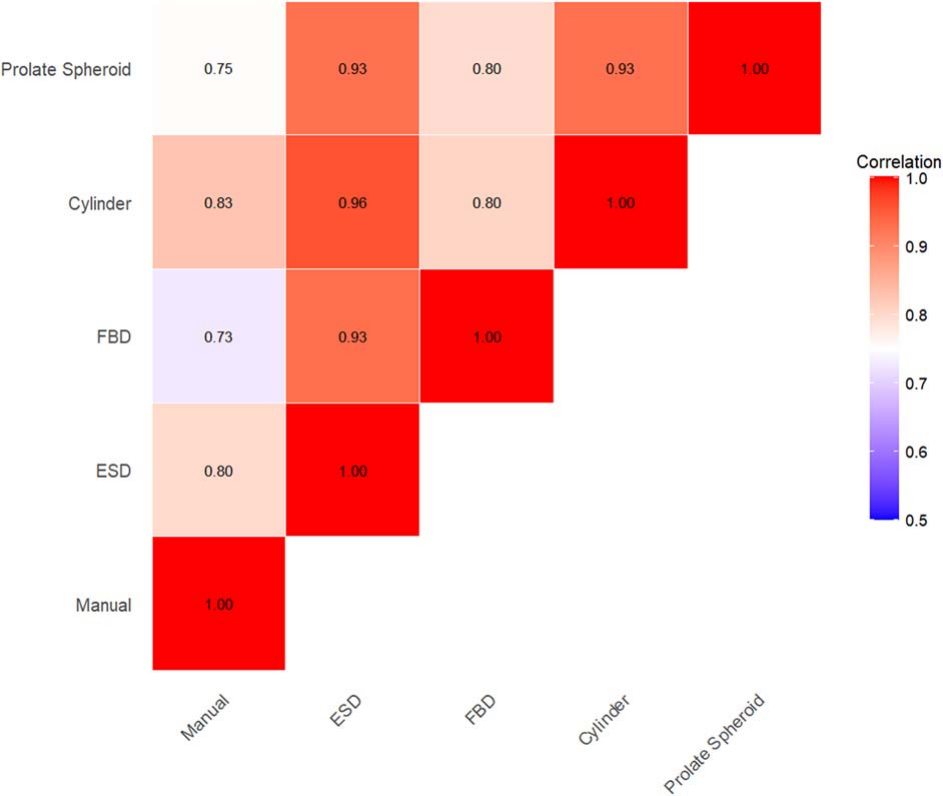
Pearson correlation matrix for biovolume estimates pooled across algal taxa and samples determined by different biovolume methods.

**Fig. 3:**
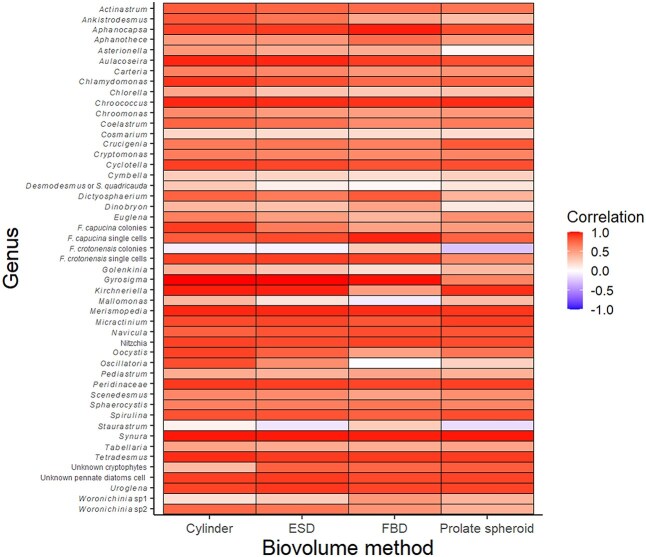
Correlation matrix for biovolume estimates of individual algal taxa determined by different biovolume methods compared to manual estimates. Numerical values are provided in Table S2.

Not surprisingly, the automatic biovolume measurements, which were pooled across all algal taxa and samples, were strongly correlated with the manual shape-based biovolume estimates and one another (Fig. 2). Of the automatic methods, the cylinder biovolume method had the strongest correlation with the manual estimates (r = 0.83). In contrast, the Feret-based diameter (FBD) biovolume had the lowest correlation with the manual estimates (r = 0.73). All biovolume measurements were moderately to strongly correlated (0.73 < r < 0.96) with one another (Fig. 2), but correlations between the automatic methods and the manual method vary within and especially among algal taxa (Fig. 3; numerical values of correlation coefficients are provided in Table S2). Whereas most correlations were strong and positive, some taxa exhibited weak or negative correlations (e.g. *Dinobryon*, *Staurastrum*, *Mallomonas*, *Golenkinia*).

### Morphology-specific biovolume estimates

**Fig. 4:**
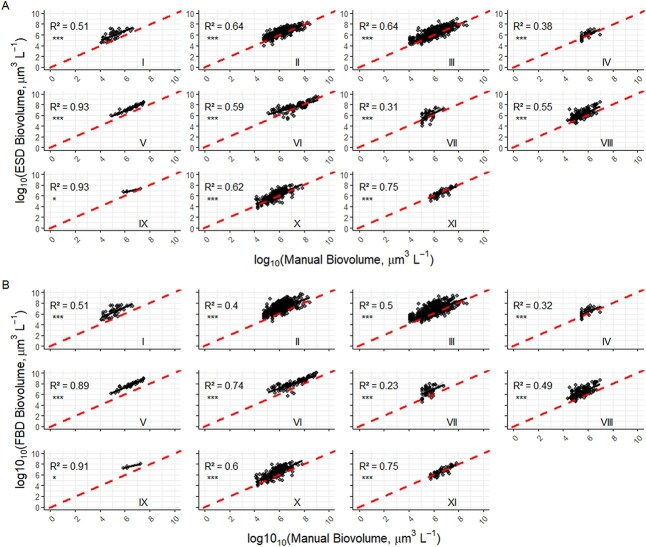
Regressions for biovolume estimates using equivalent diameters for spheres: (a) ESD biovolume; and (b) FBD volume versus manual shape-based measurement. Result are presented for different algal shape categories: (I) conical, (II) cylindrical, (III) ellipsoidal, (IV) half paralleliped, (V) irregular cluster, (VI) irregular dense, (VII) rectangular colony—cylindrical cells, (VIII) rectangular colony—ellipsoidal cells, (IX) sigmoidal, (X) spherical and (XI) spheroidal/conical. The dashed line indicates 1:1. Lighter shading indicates the 95% confidence interval, but in most cases it is not visible behind the data symbols. Asterisks indicate significant regression slopes (^*^P < 0.05, ^**^P < 0.01, ^***^P < 0.001).

After separating algal taxa into their respective shape categories (Table I), the ESD (Fig. 4a) method predicted biovolume more accurately than the FBD method, overall (Fig. 4b). Interestingly, the biovolumes of algae with cylindrical (Fig. 4 panel II), ellipsoidal (panel III) and spherical (panel X) algal morphologies were similar between the ESD and manual method but were all overestimated by FBD. Regardless, both the ESD and FBD methods had a relatively strong association (*R^2^* = 0.75) with the manual biovolume measurements for the spheroidal/conical morphologies comprised of Peridinaceaen dinoflagellates and colonial cyanobacteria with irregular clusters of cells (*R^2^* = 0.93 and *R^2^* = 0.89 for ESD and FBD, respectively). All regressions had statistically significant slopes (*P* < 0.05).

**Fig. 5:**
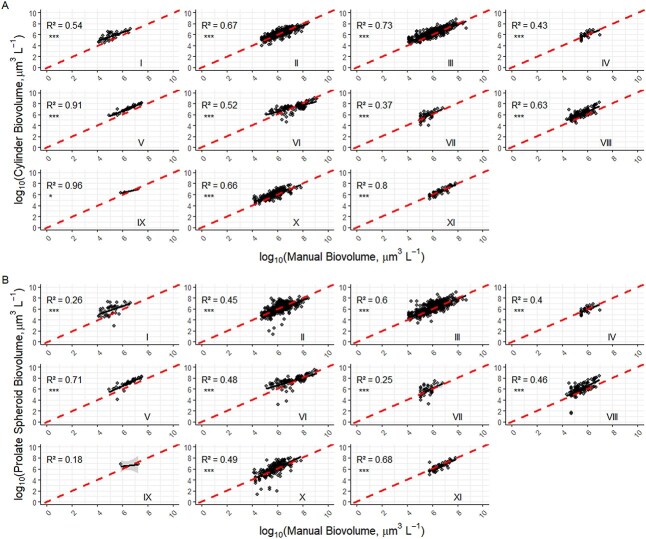
Regressions for shape-based biovolume estimates: (a) automatic cylinder biovolume method; and (b) prolate spheroid method versus manual shape-based estimates for different algal morphology categories. Panel descriptions same as Fig. 4.

Despite its intended use on cylindrical algae, the cylinder biovolume method had strong associations with the manual measurements across a variety of algal morphologies (Fig. 5a), including taxa with cylindrical morphology (panel II). In contrast, the prolate spheroid biovolume method had weaker associations with the manual method than the cylinder biovolume method for most morphologies (Fig. 5b), including the ellipsoid (panel III), which is the general form of a prolate spheroid. Furthermore, the prolate spheroid method severely underestimated the manual biovolume measurements in several samples (see Fig. 5 panels II, VI and X).

**Fig. 6:**
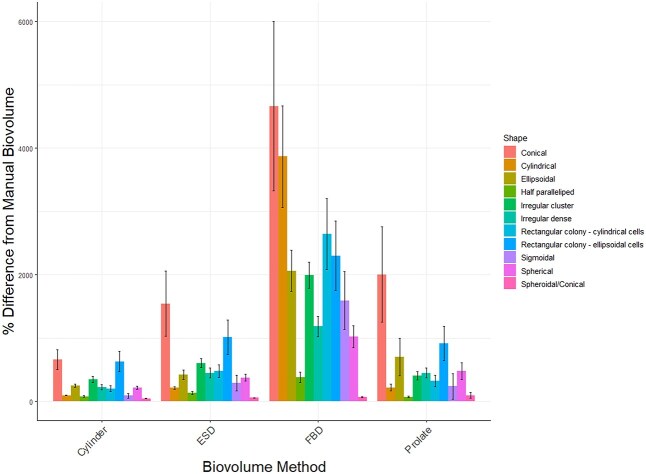
Mean difference between the biovolumes calculated by each method from the manual estimate for 11 algal shape categories. Error bars are s.e.m.

Whereas most of the regression slopes were approximately consistent with the 1:1 line slopes, the manual biovolume measurements were overestimated by the automatic methods for almost all algal morphologies and biovolume methods (Fig. 6). The FBD biovolume estimate had the largest difference from the manual method across all phytoplankton morphologies (F_3,6 656_ = 52, *P* < 0.001) and overpredicted most manual biovolume measurements by up to 5000%. The other methods did not differ significantly from the manual measurements (*P* > 0.05). Compared to FBD, the cylinder and prolate spheroid biovolume methods both had smaller differences (*P* < 0.001) from the manual measurements for cylindrical and ellipsoidal phytoplankton. There was a significant interaction between morphology and biovolume method (F_30,6 656_ = 1.92, *P* < 0.01) whereby the biovolumes of cylindrical taxa had smaller differences from the manual estimate than ellipsoidal taxa for every method except for FBD.

### Community-wide assessment

**Fig. 7:**
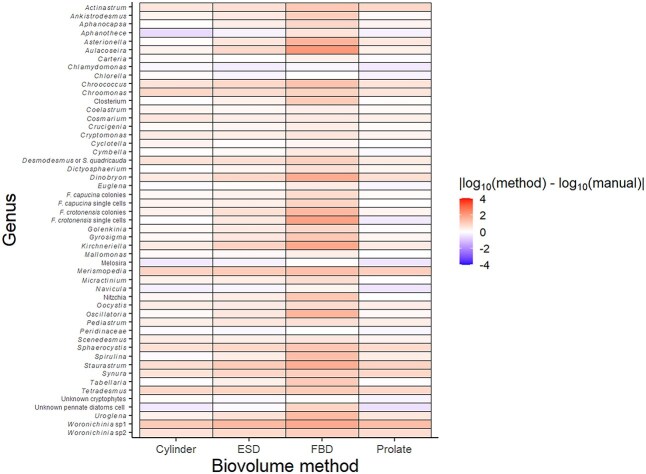
Average difference between the biovolumes calculated by each method and the manual measurements for individual algal taxa. Note that differences are on a log_10_ scale, which correspond to orders of magnitude.

The magnitude of the difference between the manual biovolume measurement and the estimate from each method also varied among and within phytoplankton taxa (Fig. 7). For most taxa, the biovolumes estimated by the automatic methods were within one order of magnitude of the manual measurements. The estimates for some algal taxa, however, over- or underestimated the manual measurements by two or more orders of magnitude. For example, the biovolumes of *Aulacoseira*, *Dinobryon*, *Staurastrum* and *Woronichinia* sp1 were severely overestimated by at least one method, usually the FBD or prolate spheroid method. Interestingly, the frequency and magnitude by which biovolumes were underestimated were lower than those that were overestimated. Example taxa include *Melosira* and *Aphanothece*. Notably, all the taxa with over- and underestimated biovolumes listed above either have complex morphologies at a cellular level (e.g. *Staurastrum*) or are colonial.

**Fig. 8:**
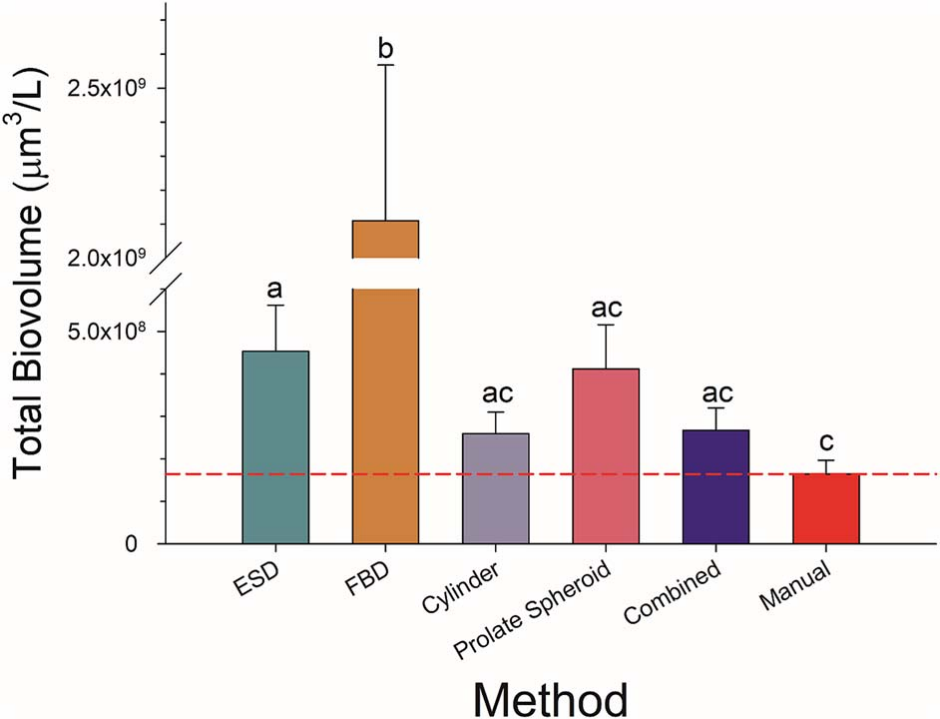
Mean total biovolume of the algal community for each method. The combined method is based on the most accurate method for specific algal morphology categories. The dashed line indicates the manual total biovolume estimate. Letter codes indicate significant differences among biovolume estimates. Error bars are standard errors.

Significant differences among mean total biovolume estimates for the algal community were found depending on the method used (F_5, 246_ = 72, *P* < 0.001; Fig. 8). All methods overestimated manual total biovolume, but FBD and ESD differed significantly (FBD: *P* < 0.001; ESD: p = 0.03) from the manual measurements. Among the automatic methods, the cylinder method had the smallest difference from the manual total biovolume measurement (59% on average). We also combined methods and used the most accurate method for each algal shape category within the community, but did not observe a significant improvement in the estimate of total biovolume (Fig. 8). Not surprisingly, the cylinder method was applied most frequently in the combined method and was applicable to an assortment of algal morphologies (Table S3). Interestingly, cylindrical cells only comprised 15% of the total biovolume in the samples. The high applicability of the cylinder method appears to be due to its relatively high accuracy for ellipsoidal cells and irregular dense colonies, which together comprised 77% of the total biovolume, rather than being due to a dominance by cylindrical algae in the samples.

## DISCUSSION

The accuracy of automatic image-based biovolume measurements depends on the selection of the algorithm used to estimate biovolume and the morphology of the phytoplankton taxon. Surprisingly, the cylinder biovolume method was the most accurate algorithm and was versatile across a broad range of algal morphologies, potentially because it depends on geodesics rather than Feret length measurements. Geodesics incorporate the area and perimeter of the particle in their calculation but also account for the curvature of the particle (Spaulding *et al.,* 2012), which may allow for particles with complex morphologies to be measured accurately. Moreover, the cylinder is one of the most common cell shapes in phytoplankton communities, especially those with high proportions of diatoms (Ryabov *et al.,* 2021; Stanca et al., 2013). In the samples analyzed in the present study, diatoms were most abundant in the early summer but comprised a moderate percentage of biovolume throughout the year (Farrow *et al.,* 2020), which partly explains the relatively high accuracy of the cylinder biovolume algorithm. The utility of cylinder method, however, is not solely linked to the frequency of cylinders in the sample, rather the cylinder method was also applicable to the high proportions of ellipsoidal cells and irregular dense colonies in the samples in our combined method (Table S3). This was also evident in a regression of the residuals from Fig. 5a vs. the proportion of diatoms in the sample, which only explained 3% of the variation in the data (data not provided).

It is relevant to note that based on previous studies, it was expected that ESD biovolume would be more accurate than FBD biovolume for most morphologies (Jakobsen and Carstensen, 2011; Hrycik *et al.,* 2019). This was due in part because area and perimeter-based measurements are less variable than linear dimensions (i.e. Feret length measurements) (Saccà, 2017). We, however, observed a consistent pattern whereby FBD overpredicted biovolumes by approximately an order of magnitude and was less accurate than any other method. In this case, the ABD and shape-based automated methods were better, especially the cylinder biovolume as discussed above.

The community-wide assessment provided insights into the mechanisms by which the accuracy of automatic image-based total biovolume estimates, and consequently, how derived productivity metrics (LaBaugh, 1995) may vary in relation to taxonomic composition. Interestingly, the biovolumes of rectangular colonies with cylindrical cells (*Fragilaria crotonensis* and *Fragilaria capucina*) and ellipsoidal cells (*Merismopedia, Desmodesmus, Scenedesmus*), as well as several taxa (e.g. *Dinobryon*, *Staurastrum*, *Mallomonas*, *Golenkinia*) with complex morphologies were not measured accurately with any method. Regardless of the shape used for manual biovolume calculations, these taxa all have at least one of the following morphological features: (i) spiny processes extending from the particle; (ii) transparent structures that may not have enough pixel intensity to be distinguished from the background of the image consistently; or (iii) cells belong to a colony with only a small number of layers and potentially a void space between individual cells. *Dinobryon* is an example that has all of these three issues because it has a branching colonial structure with transparent loricae, which encapsulate the individual ellipsoidal cells. Others have also found that *Dinobryon* cannot be measured accurately using the automatic algorithms built into imaging flow cytometers (Hrycik *et al.,* 2019; Gifford *et al.,* 2024). Proper biovolume estimates exclude extraneous extracellular features, which would otherwise cause cell volumes to be overestimated (Olenina *et al.,* 2006; Brierley *et al.,* 2007). For this reason, imaging flow cytometer-based biovolume estimates tend to overestimate total biovolumes derived from microscopy, especially when mucilage or sheath-bearing taxa are abundant (Gifford *et al.,* 2024). Moreover, rotational symmetry is important for shape-based biovolume estimates (Saccà, 2017), but even when it is present at the level of individual cells (e.g. *Dinobryon—*ellipsoidal cells), it may not be observed at the whole-colony level.

Given that the most accurate automatic biovolume estimate (cylinder) overpredicted total biovolume by 59%, there is likely some room for improvement in these relatively simple image-based biovolume methods. For example, Owen *et al.* (2022) raised concerns that the FlowCam does not calculate cylinder biovolumes correctly. Despite these concerns, the default cylinder biovolume formula was the most accurate of the automatic methods used in the present study. Whereas modifications to the cylinder biovolume formula suggested by Owen *et al.* (2022) for centric diatoms explain 1–2% more variation in regressions for cylindrical algae specifically, the modified equations did not explain more variation in the total biovolume data in the present study (data not presented).

The manual method used in the present study differs from microscopy-based studies (Álvarez *et al.,* 2012; Hrycik *et al.,* 2019; Gifford *et al.,* 2024). Specifically, our manual biovolume measurements were derived from the FlowCam and we do not have microscopy estimates to compare against the automatic estimates. It is important to note, however, that our manual biovolume estimates properly omitted extraneous extracellular features and the manual measurements were based on methods described for microscope images (Olenina *et al.,* 2006; Brierley *et al.,* 2007). Given that the accuracy of FlowCam cell measurements is comparable to those taken under light microscopes (Spaulding *et al.,* 2012), a more realistic limitation of the present study is that the contribution of certain algal shapes was not uniform across samples. The practical implication of this is that our findings for total biovolume depend on the taxonomic and morphological composition of the Nottawasaga Bay phytoplankton community and may be more comparable with other studies on temperate oligotrophic lake systems with similar community compositions (e.g. Hrycik *et al.,* 2019) than other systems.

## CONCLUSIONS

Image-based biovolume estimates depend on algal morphology, such that the accuracy of total biovolume estimates is a function of the diversity of morphologies represented in an algal community. The general diameter-based biovolume approximations (e.g. FBD and ESD) should be avoided over shape-specific biovolume approximations. Our results show that the cylinder may be used as a canonical shape for automatic image-based biovolume estimates, especially in algal communities with a large proportion of elongated shapes. When biovolume estimates are determined at the whole colony level, which is typical in imaging flow cytometry studies (Álvarez *et al.,* 2012; Hrycik *et al.,* 2019), such a simplified shape representation may provide adequate results when microscopy-based estimates are unavailable but may overpredict total biovolume by roughly 60%. We believe that the cylinder automatic image-based biovolume provides the most accurate method for biovolume estimation when the algal shapes present in a sample are unknown.

## Supplementary Material

Supplementary_materials_fbag011

## Data Availability

Data are available from the corresponding author upon request.
