# Supplementary material for: Effect of phytoplankton morphology on the measurement of biovolume
Source: J Plankton Res. 2026 Mar 5;48(2):fbag011. doi: 10.1093/plankt/fbag011 (PMC13016873; doi:10.1093/plankt/fbag011)
Supplement: Supplementary_materials_fbag011 [file supplementary_materials_fbag011.docx]

**Supplemental files for Farrow and Ackerman, Effect of phytoplankton morphology on the measurement of biovolume**

**Tables S1, S2, and S3**

Table S1. Average dimensions and manual biovolume measurements for cells of each genus. Dimensions and biovolumes for *Oscillatoria*, *Pediastrum*, and *Spirulina* are reported for the entire filament/colony. Asterisks indicate taxa whose heights were estimated using ratios from Olenina et al., (2006) and Napiorkówska-Krzebietke and Kobos (2016). As necessary, the heights for other taxa were approximated as being equal to diameter or two thirds the diameter. Dashes (—) indicate cases where a dimension is not applicable.

| Genus | Average Length (µm) | Average Diameter (µm) | Average Height (µm) | Average Cell/Colony Biovolume (µm^3^) |
| --- | --- | --- | --- | --- |
| *Asterionella* | 55.0 | 4.6 | 4.6 | 1143.4 |
| *Aulacoseira* | 21.3 | 8.1 | — | 1091.6 |
| *Carteria* | 13.4 | 9.0 | — | 569.2 |
| *Actinastrum* | 16.8 | 3.2 | — | 45.0 |
| *Chlorella* | — | 7.2 | — | 193.5 |
| *Chroococcus* | — | 7.3 | — | 205.8 |
| *Merismopedia* | 4.5 | 3.5 | — | 28.0 |
| *Oscillatoria* (filament) | 63.9 | 6.0 | — | 1790.1 |
| *Cryptomonas* | 25.1 | 10.1 | 6.7 | 895.9 |
| *Chroomonas* | 12.5 | 5.5 | 3.7 | 132.6 |
| *Closterium* | 134.8 | 20.5 | — | 14889.6 |
| *Coelastrum* | — | 7.2 | — | 197.0 |
| *Crucigenia* | 14.6 | 8.4 | — | 544.7 |
| *Cyclotella** | — | 10.5 | 6.8 | 588.3 |
| *Cymbella* | 43.9 | 12.2 | 12.2 | 3272.7 |
| *Desmodesmus* | 12.5 | 4.5 | — | 131.8 |
| *Dictyosphaerium* | — | 8.8 | — | 352.6 |
| *Dinobryon* | 11.0 | 5.9 | — | 198.3 |
| *Euglena** | 49.0 | 14.3 | 11.4 | 4194.9 |
| *F. capucina* | 31.7 | 4.3 | 4.3 | 581.3 |
| *F. crotonensis* single cell | 148.4 | 3.7 | 3.7 | 2081.7 |
| *F. crotonensis* cells within colonies | 92.4 | 3.5 | 3.5 | 1156.1 |
| *Golenkinia* | — | 16.9 | — | 2505.9 |
| *Melosira* | 24.4 | 17.3 | — | 5751.0 |
| *Oocystis* | 10.7 | 6.8 | — | 259.4 |
| *Navicula** | 40.7 | 8.5 | 8.5 | 2943.5 |
| *Nitzschia* | 47.2 | 4.3 | 4.3 | 880.6 |
| *Scenedesmus* | 12.7 | 5.0 | — | 165.3 |
| *Sphaerocystis* | — | 6.6 | — | 149.3 |
| *Tabellaria* | 67.3 | 9.2 | 9.2 | 5646.4 |
| *Uroglena* | 9.1 | 6.0 | — | 173.8 |
| *Mallomonas* | 22.0 | 13.1 | — | 1983.7 |
| *Gyrosigma** | 102.0 | 13.1 | 7.9 | 7344.3 |
| *Pediastrum* (colony) | — | 34.7 | 4.9 | 4640.8 |
| *Synura* | 12.5 | 9.4 | — | 578.5 |
| *Spirulina* (filament) | 101.7 | 3.6 | — | 1022.8 |
| *Tetradesmus* | 19.1 | 3.7 | — | 69.4 |
| *Kirchneriella* | 23.7 | 6.8 | — | 284.9 |
| Peridinaceae* | 28.5 | 24.9 | — | 5990.7 |
| *Micractinium* | — | 6.3 | — | 129.0 |
| *Ankistrodesmus* | 36.9 | 2.8 | — | 74.0 |
| *Chlamydomonas* | 15.2 | 11.8 | — | 1117.5 |
| *Staurastrum* | 11.2 | 7.7 | — | 348.0 |
| *Cosmarium* | 15.2 | 8.2 | 4.9 | 648.4 |
| *Synedra* | 216.1 | 6.4 | 6.4 | 8879.5 |
| *Aphanocapsa* | — | 3.2 | — | 17.6 |
| *Aphanothece* | 2.3 | 1.6 | — | 3.0 |
| *Woronichinia* sp2 | 3.5 | 2.3 | — | 9.5 |
| *Woronichinia* sp1 | 5.4 | 3.9 | — | 43.0 |

Table S2. Correlation coefficients corresponding to fig. 3 in the text.

| **Genus** | **ABD** | **ESD** | **Cylinder** | **Prolate spheroid** |
| --- | --- | --- | --- | --- |
| *Actinastrum* | 0.76 | 0.74 | 0.80 | 0.70 |
| *Ankistrodesmus* | 0.69 | 0.45 | 0.81 | 0.35 |
| *Aphanocapsa* | 0.91 | 0.97 | 0.89 | 0.85 |
| *Aphanothece* | 0.54 | 0.73 | 0.52 | 0.52 |
| *Asterionella* | 0.44 | 0.43 | 0.53 | 0.04 |
| *Aulacoseira* | 0.96 | 0.90 | 0.96 | 0.85 |
| *Carteria* | 0.61 | 0.54 | 0.64 | 0.55 |
| *Chlamydomonas* | 0.84 | 0.74 | 0.93 | 0.78 |
| *Chlorella* | 0.31 | 0.29 | 0.46 | 0.31 |
| *Chroococcus* | 0.95 | 0.93 | 0.95 | 0.95 |
| *Chroomonas* | 0.52 | 0.50 | 0.60 | 0.54 |
| *Coelastrum* | 0.71 | 0.59 | 0.76 | 0.66 |
| *Cosmarium* | 0.19 | 0.19 | 0.22 | 0.19 |
| *Crucigenia* | 0.69 | 0.64 | 0.67 | 0.81 |
| *Cryptomonas* | 0.63 | 0.60 | 0.66 | 0.63 |
| *Cyclotella* | 0.88 | 0.83 | 0.90 | 0.84 |
| *Cymbella* | 0.23 | 0.19 | 0.27 | 0.25 |
| *Desmodesmus* or *S. quadricauda* | 0.08 | 0.04 | 0.29 | 0.14 |
| *Dictyosphaerium* | 0.67 | 0.80 | 0.77 | 0.40 |
| *Dinobryon* | 0.34 | 0.48 | 0.38 | 0.12 |
| *Euglena* | 0.50 | 0.39 | 0.65 | 0.57 |
| *F. capucina* cells in colonies | 0.66 | 0.48 | 0.91 | 0.52 |
| *F. capucina* single cell | 0.86 | 0.96 | 0.80 | 0.77 |
| *F. crotonensis* cells in colonies | -0.06 | 0.28 | -0.07 | -0.25 |
| *F. crotonensis* single cell | 0.90 | 0.89 | 0.89 | 0.61 |
| *Golenkinia* | 0.30 | 0.18 | 0.41 | 0.37 |
| *Gyrosigma* | 1.00 | 0.99 | 1.00 | 0.63 |
| *Kirchneriella* | 0.97 | 0.50 | 0.97 | 0.94 |
| *Mallomonas* | 0.16 | -0.09 | 0.37 | 0.35 |
| *Merismopedia* | 0.95 | 0.94 | 0.96 | 0.92 |
| *Micractinium* | 0.88 | 0.82 | 0.86 | 0.90 |
| *Navicula* | 0.83 | 0.85 | 0.78 | 0.83 |
| *Nitzchia* | 0.86 | 0.89 | 0.89 | 0.85 |
| *Oocystis* | 0.78 | 0.49 | 0.89 | 0.70 |
| *Oscillatoria* | 0.58 | -0.01 | 0.85 | 0.25 |
| *Pediastrum* | 0.40 | 0.47 | 0.43 | 0.40 |
| Peridinaceae | 0.90 | 0.88 | 0.91 | 0.89 |
| *Scenedesmus* | 0.59 | 0.49 | 0.62 | 0.58 |
| *Sphaerocystis* | 0.66 | 0.69 | 0.64 | 0.63 |
| *Spirulina* | 0.83 | 0.78 | 0.83 | 0.85 |
| *Staurastrum* | -0.11 | 0.27 | 0.07 | -0.15 |
| *Synura* | 0.98 | 0.97 | 0.98 | 0.98 |
| *Tabellaria* | 0.44 | 0.43 | 0.48 | 0.47 |
| *Tetradesmus* | 0.91 | 0.91 | 0.94 | 0.91 |
| Unknown cryptophytes | 0.78 | 0.75 | 0.36 | 0.80 |
| Unknown pennate diatoms cell | 0.91 | 0.87 | 0.90 | 0.88 |
| *Uroglena* | 0.92 | 0.88 | 0.88 | 0.88 |
| *Woronichinia* sp1 | 0.27 | 0.54 | 0.17 | 0.40 |
| *Woronichinia* sp2 | 0.69 | 0.55 | 0.75 | 0.41 |

Table S3. The percentage of each method applied to each algal morphology category in the combined method and the relative biovolume comprised by each cell shape in the underlying samples. The value for the most frequent method for each cell shape is bolded.

| Shape | ESD (%) | FBD  (%) | Cylinder  (%) | Prolate spheroid  (%) | Relative biovolume of shape (%) |
| --- | --- | --- | --- | --- | --- |
| (A) Irregular cluster | 0.0 | 0.0 | **61.9** | 38.1 | 1.5 |
| (B) Irregular dense | 13.7 | 6.0 | **59.8** | 20.5 | 60.6 |
| (C) Rectangular colony with ellipsoidal cells | 7.8 | 5.7 | 39.0 | **47.5** | 0.6 |
| (D) Cylindrical | 24.9 | 6.6 | 32.8 | **35.7** | 15.1 |
| (E) Half paralleliped | 11.4 | 22.9 | 20.0 | **45.7** | 0.2 |
| (F) Rectangular colony with cylindrical cells | 14.6 | 14.6 | 29.2 | **41.7** | 0.2 |
| (G) Sigmoidal | 25.0 | 0.0 | 25.0 | **50.0** | 0.1 |
| (H) Conical | 9.3 | 0.0 | **72.1** | 18.6 | 0.1 |
| (I) Ellipsoidal | 14.3 | 9.6 | **40.4** | 35.7 | 16.4 |
| (J) Spherical | 10.6 | 9.7 | **53.6** | 26.1 | 2.3 |
| (K) Spheroidal/Conical | 3.7 | 14.8 | **50.0** | 31.5 | 2.9 |
